# Supplementary material for: First and second trimester ultrasound in pregnancy: A systematic review and metasynthesis of the views and experiences of pregnant women, partners, and health workers
Source: PLoS One. 2021 Dec 14;16(12):e0261096. doi: 10.1371/journal.pone.0261096 (PMC8670688; doi:10.1371/journal.pone.0261096)
Supplement: S1 Table — (PDF) [file pone.0261096.s002.pdf]

Medline serach

Search Number	Query	Hits
1	(Ultrasound* or ultrasonograph* or sonograph* or doppler or sonogram$1 or transvaginal scan*).tw.	433681
2	((f?etal or f?etus* or Antenatal or ante-natal or prenatal or pre-natal or antepartum or ante-partum or prepartal or pre-partal or antepartal or ante-partal) adj1 screening).tw.	4408
3	exp Ultrasonography, Prenatal/	33404
4	1 or 2 or 3	447787
5	(Antenatal or ante-natal or prenatal or pre-natal or maternity or pregnan* or obstetric* or midwif* or maternal or antepartum or ante-partum or prepartal or pre-partal or antepartal or ante-partal).tw.	787483
6	Pregnancy/	884418
7	maternal health services/ or prenatal care/	40247
8	Obstetrics/	22922
9	5 or 6 or 7 or 8	1170081
10	(want* or like or desire* or require* or expect* or anticipat* or view* or experience* or perspective* or perception* or opinion* or assum* or know* or understand* or encounter* or belief* or believe* or attitude* or fear* or anxi* or reaction* or worry or worrie* or feeling* or perceive* or appreciat* or satisf* or preference* or reassur* or choice*).tw.	9265930
11	(qualitative or ethnograph* or phenomenol* or grounded theory or hermeneutic* or lived experience* or symbolic interaction* or narrative* or life experience* or action research or observation$1 or focus group* or interview* or mixed method or multimethod or multi method or survey or questionnaire* or theme* or thematic or descriptive or diary or diaries or audit* or discrete choice experiment*).mp.	2604516
12	Qualitative Research/	58304
13	Interviews as Topic/	63548
14	Narration/	8545
15	"Surveys and Questionnaires"/	475558
16	Attitude to Health/	84050
17	"Attitude of Health Personnel"/	123306
18	px.fs.	1082704
19	or/11-18	3303068
20	4 and 9 and 10 and 19	3529

CINAHL search

Search Number	Query	Hits
S1	Ultrasound* or ultrasonograph* or sonograph* or doppler or sonogram* or "transvaginal scan*"	140629
S2	(f#etal or f#etus* or Antenatal or "ante-natal" or prenatal or "pre-natal" or antepartum or "ante-partum" or prepartal or "pre-partal" or antepartal or "ante-partal") N1 screening	2604
S3	(MH "Ultrasonography, Prenatal+")	10933
S4	S1 OR S2 OR S3	142762
S5	Antenatal or "ante-natal" or prenatal or "pre-natal" or maternity or pregnan* or obstetric* or midwif* or maternal or antepartum or "ante-partum" or prepartal or "pre-partal" or antepartal or "ante-partal"	351225
S6	(MH "Pregnancy")	206420
S7	(MH "Maternal Health Services")	9794
S8	(MH "Prenatal Care")	17437
S9	(MH "Obstetrics")	6122
S10	S5 OR S6 OR S7 OR S8 OR S9	351225
S11	want* or like or desire* or require* or expect* or anticipat* or view* or experience* or perspective* or perception* or opinion* or assum* or know* or understand* or encounter* or belief* or believe* or attitude* or fear* or anxi* or reaction* or worry or worrie* or feeling* or perceive* or appreciat* or satisf* or preference* or reassur* or choice*	2175989
S12	qualitative or ethnograph* or phenomenol* or "grounded theory" or hermeneutic* or "lived experience*" or "symbolic interaction*" or narrative* or "life experience*" or "action research" or observation or observations or "focus group*" or interview* or "mixed method" or multimethod or "multi method" or survey or questionnaire* or theme* or thematic or descriptive or diary or diaries or audit* or "discrete choice experiment*"	1535683
S13	(MH "Qualitative Studies")	116714
S14	(MH "Interviews")	150846
S15	(MH "Narratives")	18156
S16	(MH "Surveys")	145466
S17	(MH "Questionnaires")	408808
S18	(MH "Attitude to Health")	44748
S19	(MH "Attitude of Health Personnel")	45230
S20	(MW "PF")	463124
S21	S12 OR S13 OR S14 OR S15 OR S16 OR S17 OR S18 OR S19 OR S20	1784721
S22	S4 AND S10 AND S11 AND S21	2544

PsycINFO search

Search Number	Query	Hits
S1	Ultrasound* or ultrasonograph* or sonograph* or doppler or sonogram* or "transvaginal scan*"	7220
S2	(f#etal or f#etus* or Antenatal or "ante-natal" or prenatal or "pre-natal" or antepartum or "ante-partum" or prepartal or "pre-partal" or antepartal or "ante-partal") N1 screening	583
S3	DE "Ultrasound"	1648
S4	S1 OR S2 OR S3	7758
S5	Antenatal or "ante-natal" or prenatal or "pre-natal" or maternity or pregnan* or obstetric* or midwif* or maternal or antepartum or "ante-partum" or prepartal or "pre-partal" or antepartal or "ante-partal"	143725
S6	DE "Pregnancy"	46252
S7	DE "Prenatal Care"	3514
S8	DE "Obstetrics"	1588
S9	S5 OR S6 OR S7 OR S8	143725
S10	want* or like or desire* or require* or expect* or anticipat* or view* or experience* or perspective* or perception* or opinion* or assum* or know* or understand* or encounter* or belief* or believe* or attitude* or fear* or anxi* or reaction* or worry or worrie* or feeling* or perceive* or appreciat* or satisf* or preference* or reassur* or choice*	3201081
S11	qualitative or ethnograph* or phenomenol* or "grounded theory" or hermeneutic* or "lived experience*" or "symbolic interaction*" or narrative* or "life experience*" or "action research" or observation or observations or "focus group*" or interview* or "mixed method" or multimethod or "multi method" or survey or questionnaire* or theme* or thematic or descriptive or diary or diaries or audit* or "discrete choice experiment*"	1486380
S12	DE "Qualitative Methods"	9278
S13	DE "Interviews"	9921
S14	DE "Narratives"	20411
S15	DE "Surveys"	8888
S16	DE "Questionnaires"	20287
S17	DE "Attitudes"	27560
S18	S11 OR S12 OR S13 OR S14 OR S15 OR S16 OR S17	1502297
S19	S4 AND S9 AND S10 AND S18	582

SocINDEX search

Search Number	Query	Hits
S1	Ultrasound* or ultrasonograph* or sonograph* or doppler or sonogram* or "transvaginal scan*"	1104
S2	(f#etal or f#etus* or Antenatal or "ante-natal" or prenatal or "pre-natal" or antepartum or "ante-partum" or prepartal or "pre-partal" or antepartal or "ante-partal") N1 screening	279
S3	S1 OR S2	1353
S4	Antenatal or "ante-natal" or prenatal or "pre-natal" or maternity or pregnan* or obstetric* or midwif* or maternal or antepartum or "ante-partum" or prepartal or "pre-partal" or antepartal or "ante-partal"	50506
S5	DE "PREGNANCY"	5325
S6	DE "MATERNAL health"	469
S7	DE "PRENATAL care"	1352
S8	DE "OBSTETRICS"	913
S9	S4 OR S5 OR S6 OR S7 OR S8	50506
S10	want* or like or desire* or require* or expect* or anticipat* or view* or experience* or perspective* or perception* or opinion* or assum* or know* or understand* or encounter* or belief* or believe* or attitude* or fear* or anxi* or reaction* or worry or worrie* or feeling* or perceive* or appreciat* or satisf* or preference* or reassur* or choice*	1145421
S11	qualitative or ethnograph* or phenomenol* or "grounded theory" or hermeneutic* or "lived experience*" or "symbolic interaction*" or narrative* or "life experience*" or "action research" or observation or observations or "focus group*" or interview* or "mixed method" or multimethod or "multi method" or survey or questionnaire* or theme* or thematic or descriptive or diary or diaries or audit* or "discrete choice experiment*"	498314
S12	DE "QUALITATIVE research"	15745
S13	DE "INTERVIEWING"	20039
S14	DE "NARRATIVE paradigm theory"	53
S15	DE "ATTITUDE (Psychology)"	22440
S16	S11 OR S12 OR S13 OR S14 OR S15	512081
S17	S3 AND S9 AND S10 AND S16	152

LILACS search

Search Number	Query	Hits
	(tw:(Ultrasound* OR ultrasonograph* OR sonograph* OR doppler OR sonogram* OR transvaginal scan*)) AND (tw:(Antenatal OR ante-natal OR prenatal OR pre-natal OR maternity OR pregnan* OR obstetric* OR midwif* OR maternal OR antepartum OR ante-partum OR prepartal OR pre-partal OR antepartal OR ante-partal)) AND (tw:(want* OR like OR desire* OR require* OR expect* OR anticipat* OR view* OR experience* OR perspective* OR perception* OR opinion* OR assum* OR know* OR understand* OR encounter* OR belief* OR believe* OR attitude* OR fear* OR anxi* OR reaction* OR worry OR worrie* OR feeling* OR perceive* OR appreciat* OR satisf* OR preference* OR reassur* OR choice*))	253

AIM search

Search Number	Query	Hits
	(tw:(Ultrasound* OR ultrasonograph* OR sonograph* OR doppler OR sonogram* OR transvaginal scan*)) AND (tw:(Antenatal OR ante-natal OR prenatal OR pre-natal OR maternity OR pregnan* OR obstetric* OR midwif* OR maternal OR antepartum OR ante-partum OR prepartal OR pre-partal OR antepartal OR ante-partal)) AND (tw:(want* OR like OR desire* OR require* OR expect* OR anticipat* OR view* OR experience* OR perspective* OR perception* OR opinion* OR assum* OR know* OR understand* OR encounter* OR belief* OR believe* OR attitude* OR fear* OR anxi* OR reaction* OR worry OR worrie* OR feeling* OR perceive* OR appreciat* OR satisf* OR preference* OR reassur* OR choice*))	16
