# Supplementary material for: First and second trimester ultrasound in pregnancy: A systematic review and metasynthesis of the views and experiences of pregnant women, partners, and health workers
Source: PLoS One. 2021 Dec 14;16(12):e0261096. doi: 10.1371/journal.pone.0261096 (PMC8670688; doi:10.1371/journal.pone.0261096)
Supplement: Attachment — Submitted filename: Reviewer Recommendation and Comments for Manuscript Number PONE.pdf [file pone.0261096.s003.pdf]

Reviewer Recommendation and Comments for Manuscript Number PONE-D-21-29907

First and second trimester ultrasound in pregnancy: a systematic review and metasynthesis of the views and experiences of pregnant women, partners, and health workers

This is a systematic review article that deals with the perception of the different actors involved in ultrasound examinations during pregnancy.

The article is well organized and has the necessary detail scans for its complete understanding.

The essay has very clear and easy-to-read language.

The reasons for the study are well presented and adequately demonstrate its relevance.

The objectives of the study are explicit and are consistent with the problem raised.

The methodology used is described in detail and seems to me correct.

The proposed methodology is consistent with the objectives and is described with the necessary detail for its understanding and analysis.

The results are presented in an organized and coherent manner.

The conclusions (discussion) of the study are compatible with the objectives and results.

Some minor details in the essay should be reviewed

In line 160, the name of the application used should be checked (EXEL or EXCEL?)

On line 311, we have a redundancy in the drafting
