# Supplementary material for: First and second trimester ultrasound in pregnancy: A systematic review and metasynthesis of the views and experiences of pregnant women, partners, and health workers
Source: PLoS One. 2021 Dec 14;16(12):e0261096. doi: 10.1371/journal.pone.0261096 (PMC8670688; doi:10.1371/journal.pone.0261096)
Supplement: Attachment — Submitted filename: Response to reviewers.docx [file pone.0261096.s004.docx]

**Response to reviewers**

Dear reviewers and editor,

Thank you for reviewing our submission and highlighting the errors raised.

We have made the changes as suggested:

- On line 160, the name of the application has been changed from EXEL to EXCEL
- On line 311, the duplicated word has been removed

We have also addressed the additional requirements:

- The PLOS ONE style requirements, including those for file naming have been checked
- The reference list has been checked to ensure that it is complete and correct

Thank you again for highlighting these errors.

Yours sincerely,

Gill Moncrieff
